# Alcohol and Hepatitis C

**Published:** 2001

**Authors:** Charles S. Lieber

**Affiliations:** Charles S. Lieber, M.D., M.A.C.P., is chief of the Section of Liver Disease & Nutrition, Alcohol Research Center, Bronx, NY Medical Center and professor of medicine and pathology at Mt. Sinai School of Medicine, New York, New York

**Keywords:** hepatitis C virus, chronic AODE (effects of alcohol or other drug use, abuse, and dependence), disease course, ethanol metabolism disorder, oxidative stress, fibrosis, hepatocyte, carcinoma, anti-infective agents, interferon, antioxidants, patient compliance

## Abstract

Infection with the hepatitis C virus (HCV) has become a leading cause of scarring of the liver (i.e., fibrosis) and cirrhosis in the United States. HCV-related cirrhosis (with its associated complications, such as liver cancer) is a major cause of death, although it develops slowly and occurs only in approximately one-third of HCV-infected patients. Alcohol can exacerbate HCV infection and the associated liver damage by causing oxidative stress and promoting fibrosis, thereby accelerating disease progression to cirrhosis. Furthermore, alcohol may exacerbate the side-effects associated with current antiviral treatment of HCV infection and impair the body’s immune defense against the virus. Of the HCV-infected people who do not consume alcohol, only a minority progresses to severe liver disease and requires antiviral treatment. Because alcohol potentiates the fibrosis- and cancer-inducing actions of HCV, alcoholics are particularly vulnerable to HCV infection and most in need of treatment.

Hepatitis is an inflammation of the liver that is characterized by jaundice, liver enlargement, abdominal and gastric discomfort, abnormal liver function, and other symptoms. Although in many patients the diseased liver is able to regenerate its tissue and retain its function, severe hepatitis may progress to scarring of the liver tissue (i.e., fibrosis), cirrhosis, liver cancer (i.e., hepatocellular carcinoma), and chronic liver dysfunction. Hepatitis can have numerous causes, such as excessive alcohol consumption or infection by certain bacteria or viruses. One common cause of hepatitis is infection with one of several types of viruses (e.g., hepatitis A, B, or C viruses). With the development of new diagnostic tools, infections with the hepatitis C virus (HCV) have received increasing attention in recent years. In the United States, the number of deaths caused by HCV is increasing and may approach or even surpass the number of deaths from AIDS in the next few years (Alter 1997, 2000).

HCV infection is becoming a leading cause of cirrhosis, liver failure, and hepatocellular carcinoma, with incidence^1^ and prevalence rates of those complications highest among nonwhite racial and ethnic groups. Lifestyle and socioeconomic factors have been implicated in these ethnic and racial differences (Howell et al. 2000). In addition to genetic factors in the infected person (Powell et al. 2000), three independent factors are associated with an increased rate of disease progression to those life-threatening consequences. These factors include daily alcohol consumption of 50 grams or more (i.e., three or more standard drinks^2^), age at infection of more than 40 years, and male gender (Ostapowicz et al. 1998). These factors have a greater influence on fibrosis progression in HCV infection than the virus itself (Poynard et al. 1997).

Early studies had reported that HCV infection (as well as infections with the hepatitis B virus) was particularly common among alcoholics, affecting 35 percent of alcohol-dependent people. However, those studies did not exclude the role of other potential risk factors, such as intravenous drug abuse and receipt of blood transfusions before 1990.^3^ To determine the association between alcoholism and HCV infection more conclusively, Rosman and colleagues (1996) screened alcoholic patients admitted for detoxification and patients attending a general medical clinic for the presence of hepatitis B and C viruses in the blood and for risk factors for infections with those viruses. The general medicine clinic patients were also screened for possible alcoholism, and those identified as nonalcoholic served as the control group for alcoholic patients who had no other known risk factors for viral hepatitis (e.g., intravenous drug use or blood transfusions). The study found that actively drinking alcoholic patients were more likely to show evidence of HCV in the blood than control patients, suggesting that alcoholism in some way is a predisposing factor for HCV infection. This conclusion is consistent with the prior observation that the presence of inflammation in the liver is strongly associated with the presence of antibodies to HCV in alcoholic patients who have no other known risk factors for the infection (Rosman et al 1993). These observations are further supported and confirmed by studies of the epidemiology and natural history of HCV infection, which are discussed in the following section.

This article explores the association between alcoholism and HCV infection in more detail. After reviewing the epidemiology and natural history of the infection, it discusses some of the mechanisms through which alcohol may exacerbate the consequences of HCV infection. The article also discusses current treatment approaches for HCV infection, particularly among drinkers.

## Epidemiology and Natural History of HCV Infection

It is estimated that HCV infects some 170 million people worldwide, and in the United States an estimated 2.7 million people have HCV infection (Lauer and Walker 2001). Nevertheless, the virus has attracted major attention among researchers and clinicians only over the last two or three decades, mainly because initially no reliable diagnostic tools were available. Moreover, the early stages of the infection are relatively benign, and severe manifestations (e.g., cirrhosis) occur only in a minority of the infected people and after long periods of time (i.e., up to 20 to 30 years) (Di Bisceglie 2000). As a result of this delay, clinicians currently note a peak of severe and life-threatening complications of HCV infection (e.g., end-stage cirrhosis and liver cancer), although maximal rates of infections may have occurred three or four decades ago.

**Figure 1:**
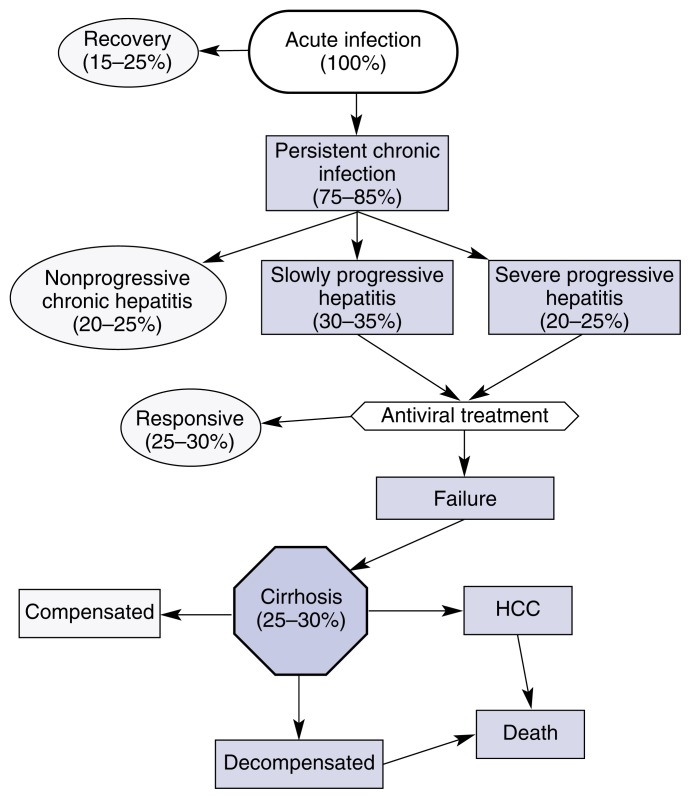
The progression of hepatitis C (HCV) infection and the proportion of initially infected patients who develop each disease stage. Approximately two-thirds of the people suffering an acute infection experience a relativity benign disease course; that is, the infection resolves on its own, does not progress, or responds to antiviral treatment. Conversely, approximately one-third of people infected with HCV develop cirrhosis, and many later die of complications from the cirrhosis (i.e., decompensated cirrhosis) or from liver cancer (i.e., hepatocellular carcinoma [HCC]). The proportions shown here provide only a general indication of how this disease progresses. The actual prognosis may vary strikingly in each patient, depending on numerous factors, including the patient’s genetic makeup; gender; age at onset of the infection; presence or absence of antiviral treatment; and, especially, concomitant alcohol use. These factors also affect the duration of disease progression, which, from the onset of the infection to the end-stages of disease, may last from 10 to 30 years.

Of the people infected with HCV, only a minority eventually develop serious, life-threatening complications (see figure 1). Thus, approximately 15 to 25 percent of infected people recover spontaneously. An additional 20 to 25 percent of infected people exhibit a stable, nonprogressive chronic hepatitis that is virtually asymptomatic and, therefore, does not require antiviral treatments, particularly because the side-effects of currently available treatments may be more severe than the symptoms of the disease itself.

In 50 to 60 percent of HCV-infected patients the disease may progress with time. Of those patients, about one-half to three-fourths show a sustained reduction in their virus levels in response to state-of-the-art antiviral treatment (i.e., a combination of the medications interferon-alpha and ribavirin) (Fried et al. 2001). The response to treatment depends on the specific strain of HCV with which the patient is infected. Six distinct HCV strains exist that differ in their genetic makeup (i.e., genotype). In the United States, the most commonly found genotypes are called 1a, 1b, 2, and 3. Among patients infected with these HCV strains, those infected with HCV genotypes 1a or 1b show lower response rates to treatment (i.e., 46 percent) than do patients infected with HCV genotypes 2 or 3 (i.e., 76 percent).

Treatment failure and, consequently, disease progression to cirrhosis, liver failure, or hepatocellular carcinoma, occurs in approximately 25 to 30 percent of patients originally infected with HCV (see figure 1). Because these patients will develop serious and potentially fatal health consequences, it is essential to devise ways to identify them before they enter the advanced stages of the disease in order to initiate early treatment and avoid those factors that promote rapid disease progression toward the end-stages.

Because of the potentially serious consequences of HCV infection, prevention is an important concern. The primary prevention approach obviously is to avoid the main sources of infection, such as intravenous drug abuse or transfusion of contaminated blood. Another prevention approach would be to avoid sexual promiscuity, which promotes acquisition of the disease (Alter 2000) in ways that have not yet been well defined. In addition to these “classic” risk factors, other factors can significantly increase the rate of infection, its persistence, or the rapid evolution of the disease toward the dismal end-stages. Although some of these factors (e.g., genetic factors, gender, and age at infection) cannot be controlled, avoidance of other factors could have a tremendous impact on the spread and incidence of HCV infection. Among these controllable risk factors, none is more important than alcohol consumption.

## Effect of Alcoholism on HCV Infection

Researchers first became aware of the major effect of alcoholism on HCV infection when they noted that alcoholism was associated with HCV (but not hepatitis B) even in people who did not show classic risk factors, such as intravenous drug abuse or blood transfusions (Rosman et al. 1996; also see Schiff 1997). In addition to promoting the acquisition or persistence of HCV, alcohol subsequently was shown to affect the two major processes that are harbingers of rapid and severe progression of liver disease and of the patient’s deterioration, namely inflammation and fibrosis.

### Effects of Alcoholism on HCV Acquisition and Persistence

**Figure 2:**
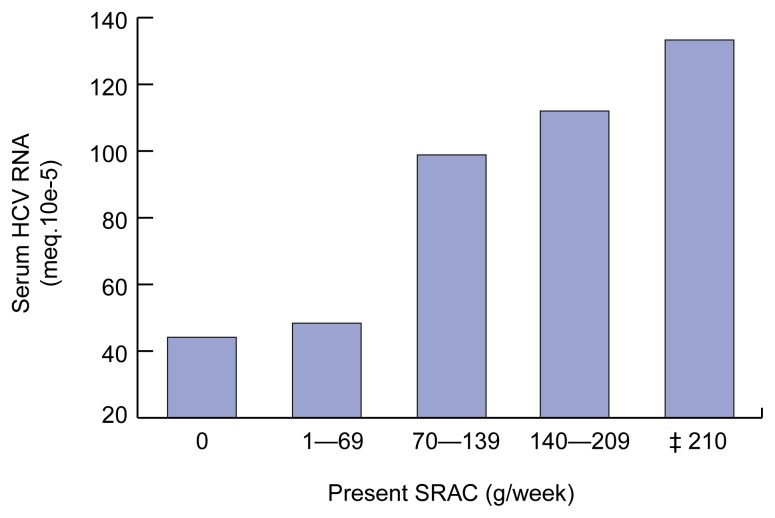
Relationship between hepatitis C virus (HCV) levels in the blood and self-reported alcohol consumption (SRAC) (in grams of alcohol per week*) during a typical week in the month preceding the HCV measurement. Greater alcohol consumption was associated with higher virus levels in the blood. *One standard drink (i.e., 12 fluid ounces of beer, 5 fluid ounces of wine, or 1.5 fluid ounces of distilled spirits) contains approximately 14 grams (0.5 ounces) of pure alcohol. NOTE: Statistical significance: *r* = 0.26, *p*<0.0001. SOURCE: Pessione et al. 1998, with permission.

In addition to the high incidence of HCV infection in heavy drinkers even in the absence of classic risk factors, other observations suggest that heavy alcohol consumption enhances the ability of the virus to enter and persist in the body. For example, several studies demonstrated a correlation between the presence of virus in the blood (i.e., viremia) and the amount of alcohol patients reported they consumed (i.e., self-reported alcohol consumption, or SRAC) (see figure 2). Furthermore, moderation of alcohol consumption was shown to result in a decrease in the number of virus particles in the blood (i.e., the viral titer) (Cromie et al. 1996). Researchers do not yet fully understand the mechanism through which alcohol affects the viral titer. It is well known, however, that alcohol impairs the function of certain components of the body’s immune system (Ince and Wands 1999). An impaired immune function, in turn, may influence the ability of the virus to persist in the body rather than be eliminated by immune cells.

Another mechanism through which alcohol consumption may favor the progression and exacerbation of HCV infection is oxidative stress. The term “oxidative stress” refers to the presence of excessive levels of highly reactive molecules called free radicals in the cell or a lack of molecules called antioxidants that can eliminate those free radicals. (For more information on oxidative stress, see the sidebar, p. 249.) Various studies have indicated that through as yet unknown mechanisms, HCV infection itself can lead to oxidative stress (Larrea et al. 1998), which contributes to the virus’s ability to persist in the body. This virus-induced oxidative stress, in turn, may be exacerbated by the breakdown (i.e., metabolism) of alcohol in the liver, which can generate free radicals that contribute to oxidative stress (Lieber 1997) and which are a major cause of alcohol-related hepatic injury (Lieber 2001).

**Oxidation and Formation of Free Radicals**

The breakdown of nutrients (e.g., carbohydrates, proteins, and fats) as well as other molecules (e.g., alcohol) frequently involves chemical reactions that use oxygen and/or hydrogen (i.e., oxidation reactions). Generally speaking, oxidation reactions are those that add oxygen to or remove hydrogen from a substance (or both). For example, the metabolism of alcohol involves two oxidation reactions. First, one enzyme converts alcohol (chemically referred to as ethanol) to acetaldehyde by removing hydrogen. Then, a second enzyme converts acetaldehyde to acetate by removing additional hydrogen and adding oxygen.

Two major enzyme systems are involved in ethanol metabolism in the liver. The first one involves the enzyme alcohol dehydrogenase. The second system, which is activated mainly after heavy alcohol consumption, is the microsomal ethanol-oxidizing system (MEOS). Particularly the MEOS, however, sometimes generates not only stable, nontoxic molecules but also highly unstable (i.e., reactive) and potentially harmful molecules, called free radicals. Many of these molecules contain oxygen and are called oxygen radicals. Common oxygen radicals include superoxide (O_2_^•^), hydrogen peroxide (H_2_O_2_), and hydroxyl radicals (OH^•^). The presence of excess levels of oxygen radicals is called oxidative stress. If unchecked, oxygen radicals can damage cells by attacking vital cell components, such as the fat and protein constituents of the cell wall and the cell’s genetic material. For example, oxidative stress can induce enhanced metabolism of fat molecules (i.e., lipid peroxidation) that may generate biologically active molecules. Some of these molecules, in turn, may contribute to the development of fibrosis.

Because the formation of oxygen radicals is a natural process that occurs during many metabolic processes, cells have developed several protective mechanisms to prevent radical formation or to detoxify radicals. These mechanisms employ molecules called antioxidants, which are found in foods or generated by the body itself. Commonly found antioxidants include vitamin E, vitamin C, and glutathione (GSH). These compounds have several mechanisms of action. For example, GSH can neutralize oxygen radicals by transferring hydrogen to the reactive molecules, thus creating a more stable chemical structure.

Using their internal antioxidants, cells can deal with normal levels of oxygen radical formation. When oxygen radical formation is greater than normal or antioxidant levels are lower than normal, however, oxidative stress occurs that may contribute to cell death and tissue damage, such as fibrosis of the liver. Chronic alcohol consumption can increase oxidative stress in several ways. For example, alcohol metabolism by the MEOS is associated with the generation of oxygen radicals. Moreover, animal models demonstrated that chronic alcohol consumption reduces the levels of various antioxidants, including GSH (Colell et al. 1998; Nanji and Hiller-Sturmhöfel 1997). Accordingly, treatment with potent antioxidants or with compounds to enhance the body’s ability to generate antioxidants may relieve oxidative stress and counteract the fibrosis-inducing effects of alcohol and other conditions (e.g., infection with the hepatitis C virus).

—Susanne Hiller-Sturmhöfel

ReferencesColellASelective glutathione depletion of mitochondria by ethanol sensitizes hepatocytes to tumor necrosis factorGastroenterology115154115511998983428310.1016/s0016-5085(98)70034-4NanjiAAHiller-SturmhöfelSApoptosis and necrosis: Two types of cell death in alcoholic liver diseaseAlcohol Health & Research World214325330199715706744PMC6827678

### Alcohol Metabolism and Oxidative Stress

**Figure 3:**
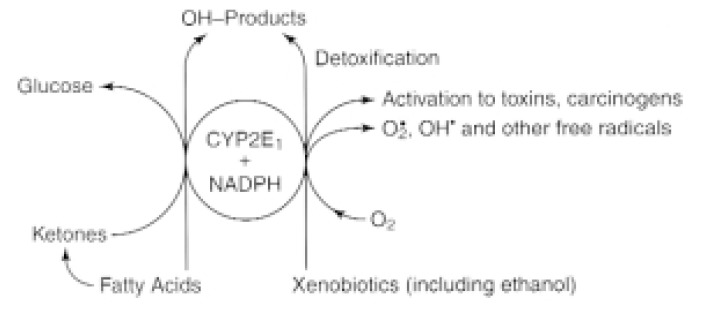
The beneficial and toxic roles of CYP2E1, an enzyme involved in the breakdown of alcohol in the liver that acts in conjunction with another compound (i.e., nicotinamide adenine dinucleotide phosphate or NADPH). CYP2E1 typically helps process compounds that are normally present in the body (e.g., fatty acids and ketones), and breaks down potentially toxic foreign substances (i.e., xenobiotics), including alcohol. Enhanced CYP2E1 activity, however, also results in the increased generation of harmful byproducts (e.g., acetaldehyde from alcohol) and other toxins, such as free radicals (e.g., superoxide [O_2_^•^] and hydroxyl [OH^•^]) that can cause liver injury by promoting excessive breakdown of fat molecules (i.e., lipid peroxidation). SOURCE: Lieber 1999*a*, with permission.

It is well known that heavy alcohol consumption can result in toxic effects on the liver (i.e., hepatotoxicity), even in people who eat a healthy diet (Lieber et al. 1965). This toxicity has been linked to alcohol metabolism in the liver (Lieber 1992). Alcohol (chemically referred to as ethanol) is broken down mainly by the enzyme alcohol dehydrogenase (ADH), which converts ethanol to acetaldehyde and hydrogen. Excess hydrogen causes a number of metabolic disorders, including fat accumulation in the liver (i.e., fatty liver) (Lieber 1995). The acetaldehyde, which itself is a toxic substance, subsequently is further metabolized by another enzyme (Lieber 1995). Acetaldehyde contributes to various toxic and metabolic effects of alcohol, but cannot account for all disorders found in alcoholics. Instead, another metabolic pathway called the microsomal ethanol-oxidizing system (MEOS) (Lieber and DeCarli 1970), which also converts ethanol to acetaldehyde, plays a role in some of alcohol’s adverse effects. The physiologic role of the MEOS is to generate the sugar glucose from various precursors; metabolize certain components of fat molecules (i.e., fatty acids); and detoxify foreign substances, including alcohol (Lieber 1999*a*) (see figure 3). Chronic alcohol consumption strongly increases the activity of the MEOS, including that of an enzyme called cytochrome P-450. Several variants of cytochrome P-450 exist, including one called CYP2E1 whose activity is markedly enhanced after chronic alcohol consumption.

In addition to its beneficial physiologic function, the MEOS can have some adverse metabolic effects (see figure 3). For example, CYP2E1 has a high capacity to break down some commonly used drugs (e.g., the over-the-counter pain medication acetaminophen [Tylenol^®^]) into toxic metabolites and to generate substances that promote the development of certain cancers. In addition, the MEOS generates toxic free radicals when it has been induced by alcohol. In patients with HCV infection, these free radicals most likely potentiate the HCV-associated oxidative stress and the resulting liver damage. This hypothesis is supported by the observation that in a clinical study, an antioxidant (i.e., vitamin E) that should reduce the level of oxidative stress improved the liver function^4^ of patients with HCV-induced liver damage (Von Herbay et al. 1997). The improvement was only partial, however, and occurred in only one-half of the patients. Therefore, researchers are currently conducting studies with more potent antioxidants, such as a substance called polyenylphosphatidylcholine (PPC), which is discussed in more detail in the section “Treatment of Hepatitis C in Drinkers.”

### Effect of Alcoholism on HCV-Induced Hepatic Inflammation

HCV infection leads to an inflammatory reaction in the liver. This inflammation is caused both by the attack of the virus on the liver cells and by the body’s defense mechanisms that are triggered by that attack. Alcohol appears to potentiate this inflammatory reaction, because HCV-infected patients who consumed alcohol exhibited greater inflammation than did patients who consumed no alcohol (Cromie et al. 1996). The exact mechanisms through which alcohol enhances hepatic inflammation remain unclear, however.

### Effects of Alcohol on HCV-Induced Fibrosis

**Figure 4:**
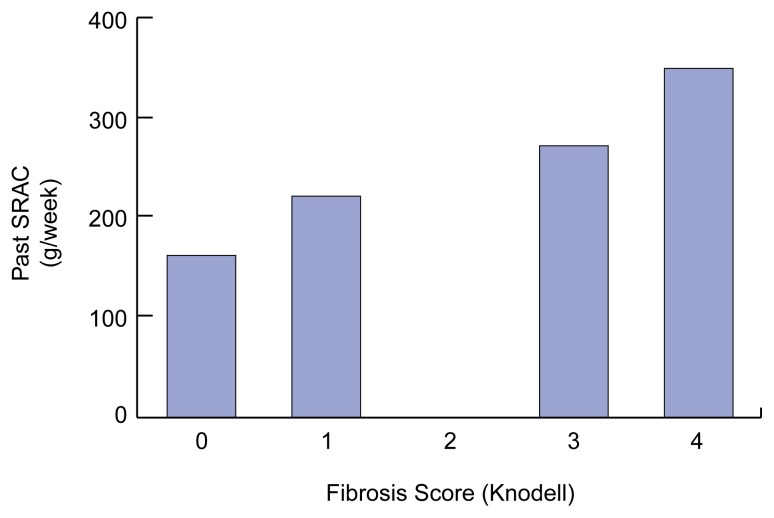
Relationship between mean self-reported alcohol consumption (SRAC) prior to the diagnosis of hepatits C virus (HCV) infection (expressed in grams per week*) and the severity of fibrosis (i.e., scarring of liver tissue, which indicates an early stage of liver disease). The severity of fibrosis was assessed using the Knodell index, which measures changes in the tissue’s structure and chemical composition (i.e., histological changes). The data show that greater alcohol consumption was associated with more severe fibrosis (i.e., greater liver damage) by the time the HCV infection was diagnosed, and therefore with more rapid disease progression. *One standard drink (i.e., 12 fluid ounces of beer, 5 fluid ounces of wine, or 1.5 fluid ounces of distilled spirits) contains approximately 14 grams (0.5 ounces) of pure alcohol. NOTE: Statistical significance: *p*<0.02 (univariate analysis). SOURCE: Pessione et al. 1998, with permission.

Several studies have shown that the rate with which HCV-induced hepatic scarring (i.e., fibrosis) progresses is significantly correlated with alcohol consumption. For example, Pessione and colleagues (1998) found that even moderate alcohol intake^5^ of approximately one to two standard drinks per day increased not only the virus levels in the blood (see figure 2) but also the extent of hepatic fibrosis (see figure 4). Other researchers detected such an acceleration in fibrosis development only with heavy alcohol consumption (i.e., approximately 3.5 standard drinks, or 50 grams of alcohol, per day) (Poynard et al. 1997). Wiley and colleagues (1998) examined the effect of long-term heavy drinking on the progression of tissue damage and clinical symptoms associated with HCV infection. The study included women who consumed more than 40 grams of alcohol (approximately 3 standard drinks) daily and men who consumed more than 60 grams of alcohol (approximately 4 standard drinks) daily for more than 5 years. The investigators concluded that alcohol intake was an independent risk factor for the progression of HCV infection. Specifically, heavy drinkers had a two- to threefold greater risk of cirrhosis and decompensated liver disease than did control subjects. Finally, Harris and colleagues (2001) found that a history of heavy alcohol abuse (i.e., more than 80 grams of alcohol, or 6 drinks, per day) was associated with a fourfold increased risk for cirrhosis. These findings that alcohol can accelerate liver damage associated with HCV infection are particularly important because HCV-infected patients generally do not become sick or die because of the presence of virus in the blood but because of the complications of the cirrhosis (see figure 1).

Alcohol influences the scarring process through several mechanisms. One of these is oxidative stress, which, as described earlier, exacerbates the oxidative stress associated with HCV itself. For example, oxidative stress can induce the excessive breakdown of fat molecules (i.e., lipid peroxidation). Some products of lipid peroxidation induced by oxidative stress have been shown to be toxic and promote fibrosis in the liver (Tsukamoto 1993). Lipid peroxidation products are detectable in the livers of patients with chronic HCV infection, especially in areas where scar tissue is being formed, suggesting a role for lipid peroxidation in HCV-associated liver fibrosis (Paradis et al. 1997).

The role of oxidative stress in fibrosis also is supported by findings that the physiologic antioxidant vitamin E is depleted in patients with alcohol-induced cirrhosis (Leo et al. 1993). Furthermore, Houglum and colleagues (1997) demonstrated that in HCV-infected patients who did not respond to treatment with interferon, vitamin E administration prevented the activation of a type of liver cell called stellate cells, whose activation plays a key role in fibrosis development. However, vitamin E treatment was only partially effective because it did not significantly affect liver function as assessed through the levels of liver enzymes in the blood, HCV virus titers, or the degree of liver inflammation. Therefore, more potent antioxidative agents are now being investigated with regard to their ability to prevent fibrosis and inflammatory reactions. (For more information on this approach, see the section on “Treatment of Hepatitis C in Drinkers.”)

### Combined Effects of HCV and Alcohol on Hepatocellular Carcinoma

Hepatocellular carcinoma almost exclusively occurs in patients who already have developed cirrhosis (see figure 1). Because HCV infection and alcohol both enhance the risk of cirrhosis, their combination results in a marked increase in the risk of cirrhosis and, consequently, in accelerated development of hepatocellular carcinoma (Tsutsumi et al. 1996). In fact, in the study by Tsutsumi and colleagues (1996), which was conducted in Japan, more than 50 percent of alcoholic HCV-infected patients developed hepatocellular carcinoma, a percentage considerably higher than that found in patients with only one or none of those two risk factors. The number of hepatocellular carcinoma cases in the United States also has increased over the past two decades because the prevalence of HCV infection has increased and with it the number of alcoholics infected with HCV. Moreover, the age-specific incidence of hepatocellular carcinoma has progressively shifted toward younger people because of more rapid disease progression (El-Serag and Mason 1999).

## Treatment of Hepatitis C in Drinkers

As mentioned earlier, approximately one-third to one-half of HCV-infected patients spontaneously recover from the infection or show only minimal disease progression with persistently normal liver function. For these patients, standard antiviral treatment (which is described in the following paragraph) is currently not recommended except as part of clinical trials, because treatment frequently is associated with severe side-effects (Shaib et al. 2000). Nevertheless, a significant number of HCV-infected patients (approximately 50 to 60 percent) are at risk for progression to severe and often fatal end-stage liver disease (Seeff 2000) and therefore should receive therapy to prevent this progression. The decision to administer antiviral treatment depends to a large extent on the physician’s ability to estimate the likelihood that a given patient will progress to more severe disease stages. In addition to genetic influences, age, and gender, multiple factors can affect the progression of fibrosis (Lieber 1999*b*). One way to determine these factors is to assess the degree of liver disease by taking a tissue sample of the liver (i.e., a liver biopsy). Such a biopsy should be obtained for all patients with demonstrated HCV viremia as well as some indication of liver disease, such as repeated findings of elevated levels of liver enzymes in the blood. Antiviral treatment is recommended for patients with HCV in the blood and findings of fibrosis and moderate inflammation on liver biopsy (National Institutes of Health Consensus Conference 1997).

Current treatment of HCV infection generally consists of a combination of the medications interferon-alpha and ribavirin. Interferon-alpha is a natural protein made in the body when cells are exposed to viruses. It induces the production of another protein that, in turn, prevents the virus from generating the proteins it needs to reproduce. Interferon-alpha has been used since 1989 to treat HCV infection; however, in many cases the patients’ response to treatment was only temporary. Subsequently, interferon-alpha was combined with the antiviral medication ribavirin, which enhanced treatment effectiveness in many patients. Nevertheless, many patients still do not respond to this combination treatment or relapse once treatment is stopped. Moreover, both interferon-alpha and ribavirin commonly have substantial side-effects, such as flulike symptoms, headache, fatigue, fever, loss of appetite, depression, insomnia, and lower-than-normal levels of various blood cell types. Because these side-effects often are more serious or bothersome to the patient than the underlying HCV infection, the treatment guidelines described in the previous paragraph were established. In addition, interferon-alpha must be administered three times per week by injection, making treatment demanding for the patient. A newly modified form of interferon-alpha called pegylated interferon-alpha has been developed, however, that need only be injected only once per week and which results in higher response rates than interferon-alpha alone.

### Alcohol Consumption in HCV-Infected Patients

Because alcohol promotes the progression of severe fibrosis to cirrhosis with severe and often fatal end-stage liver disease, the National Institute of Health Consensus Conference (1997) has stated that “more than one drink per day is strongly discouraged in patients with hepatitis C, and abstinence from alcohol is recommended.” This recommended proscription of drinking alcohol in HCV-infected patients appears justified because as mentioned earlier, some studies found that even moderate alcohol consumption may have some adverse effects on the liver in these patients. In fact, continued alcohol intake for various reasons is considered a major contraindication to therapy with interferon-alpha alone or in combination with ribavirin (McHutchison 2000). For example, interferon-alpha is known to exacerbate mental disorders (e.g., depression) that frequently occur in alcoholics. Alcohol also reduces the effectiveness of interferon-alpha treatment (Ince and Wands 1999). Finally, heavy drinkers cannot be trusted to faithfully and safely carry out a treatment program that requires them to inject themselves with interferon-alpha three times per week. (This concern might be alleviated with the introduction of pegylated interferon-alpha, which requires only one weekly injection that, if necessary, can be administered by a health worker.)

Even in HCV-infected alcoholics who stop drinking, the response to interferon-alpha is less than that in nonalcoholics (Okazaki et al. 1994), with the extent of the response depending on the level of alcohol consumption before the initiation of therapy. Thus, alcoholics who consumed less than 70 grams of alcohol per day achieved better responses than those who consumed more (Okazaki et al. 1994; Ohnishi et al. 1996). However, as mentioned earlier, the actual level of alcohol intake that significantly promotes hepatic fibrosis in HCV-infected patients still is unknown. And although abstinence obviously is preferable even to light drinking in HCV-infected patients, many heavy drinkers often cannot sustain this goal. For these reasons, and in view of the potentially severe effects of the combination of HCV infection and alcohol, even less-than-optimal antiviral treatment in alcoholic patients may be preferable to no treatment at all. This is especially true for those patients whose alcohol consumption can be reduced to a light or moderate level. A greater number of clinical studies are needed to settle this issue, however. In any event, complete cessation of alcohol intake, or at least a reduction to moderate levels, is crucial in HCV-infected patients and should receive the highest priority when treating these patients.

### The Role of Antioxidants in the Treatment of HCV Infection

As mentioned previously, oxidative stress caused both by HCV itself and by concurrent alcohol consumption plays an important role in the acquisition, persistence, and progression of HCV infection. Accordingly, antioxidant therapy to reduce oxidative stress is being investigated for the treatment of HCV infection and its associated consequences. The body’s natural defense mechanism against oxidative stress in the liver involves an antioxidant called glutathione (GSH), which detoxifies the free radicals (also see sidebar, p. 249). Various attempts have been made to boost this defense system (for reviews, see Bonkovsky 1997; Lieber 1997), including replenishment of the GSH consumed by the oxidative stress. GSH is a molecule that consists of three amino acids, including cysteine. The body’s supply of cysteine is relatively low, limiting GSH production. In a pilot study, administration of a modified version of cysteine led to an improved response to interferon-alpha in patients with chronic HCV infection (Beloqui et al. 1993). No such studies have been conducted in alcoholic HCV-infected patients, however.

Another source of decreased levels of GSH in the liver and enhanced oxidative stress are excess levels of “free iron” (i.e., iron that is not bound to other molecules) in the liver. Chronic alcohol consumption can substantially increase iron levels in the body (see Nanji and Hiller-Sturmhöfel 1997), thereby increasing oxidative stress. One approach to reduce the levels of free iron in the body is to draw a substantial amount of blood from the patient (i.e., perform a phlebotomy). Because red blood cells require iron for their function, such a blood loss will stimulate new blood cell production, which in turn will use some of the excess iron. In HCV patients, however, therapeutic phlebotomy has been associated only with biochemical improvement (i.e., improved liver function as indicated by reduced levels of liver enzymes in the blood), but not with virological improvement (i.e., reduced virus levels in the body) (Bonkovsky 1997). Accordingly, an obvious need exists for a more effective, yet safe, antioxidant therapy.

Investigators currently are conducting studies with the antioxidant PPC, which is a mixture of chemicals called polyunsaturated phosphatidylcholines that are extracted from soybeans. In baboons, PPC resulted in the complete arrest or prevention of alcohol-induced bands of fibrosis in the liver (i.e., septal fibrosis) as well as of cirrhosis (Lieber et al. 1994). PPC treatment also reduced the alcohol-induced oxidative stress in animals (Lieber et al. 1997). Oxidative stress, as mentioned earlier, may induce lipid peroxidation, resulting in the generation of molecules that can induce fibrosis. Thus, by decreasing oxidative stress, PPC may reduce lipid peroxidation, thereby preventing the generation of fibrosis-inducing molecules.

A study in HCV-infected patients found that PPC improved liver function as indicated by reduced levels of liver enzymes in the blood (Niederau et al. 1998). During that study, no liver biopsies were performed, however, and it is therefore unknown whether PPC also improved HCV-associated fibrosis. Such information is crucial, however, because as mentioned earlier, HCV-infected patients generally do not die of the virus infection per se but of the complications of the associated fibrosis and cirrhosis, including hepatocellular carcinoma (see figure 1). Therefore, a major task for the future is to implement antifibrotic treatments that can be safely combined with the antiviral therapy. PPC appears to be a promising agent, because at least in experimental animals it had both antioxidant and antifibrotic effects. Such a combined antioxidative and antifibrotic activity would be particularly indicated in HCV-infected patients who also drink because these patients are most vulnerable and desperately need a treatment that is not only effective, but also devoid of significant side-effects. PPC to date has shown no side-effects, and investigators are currently assessing its effectiveness in combination with antiviral agents in HCV-infected patients who also drink. Other antifibrotic agents (e.g., pentoxifylline, silymarin, and colchicine) were shown to have some effect in various experimental models of fibrosis, but have not yet been evaluated in HCV-infected drinkers treated with antiviral agents.

## Conclusions

HCV infection is particularly common in alcoholics, and the disease course and rate of progression can be exacerbated by alcohol consumption. Therefore, the reduction or complete cessation of alcohol consumption should be a primary focus of the treatment of HCV-infected patients. In addition, there is a persistent need for novel effective and safe treatments to arrest or reverse disease progression to the dismal end-stages. Various antioxidant and antifibrotic agents have shown promise in experimental animals, and some of these are being studied in HCV-infected humans. If the promising findings of the animal studies can be replicated in humans, such agents could considerably improve the prognosis of all HCV-infected patients, particularly HCV-infected drinkers who are at greatest risk for the most serious complications associated with the infection.

## Footnotes

1 For a definition of this and other technical terms used in this article, see the glossary, p. 253.

2 A standard drink is defined as 12 fluid ounces (oz) of regular beer, 5 fluid oz of wine, or 1.5 fluid oz of distilled spirits (80 proof) and contains approximately 0.5 oz (14 grams) of pure alcohol (Dufour 1999).

3 Before 1990, no reliable tests for detecting HCV in the blood were available, leading to a risk of HCV infection through transfusion of contaminated blood. Since 1990, the introduction of improved blood screening tests has substantially reduced the risk of transfusion-related HCV infection (Lauer and Walker 2001).

4 Liver function in this and many other studies was assessed by measuring the levels of an enzyme called aminotransferase in the patients’ blood. Elevated blood levels of this enzyme, which normally is found in the tissues, including the liver, indicate acute liver disease.

5 The National Institute on Alcohol Abuse and Alcoholism (NIAAA) classifies drinkers as light if they consume 1–13 standard drinks per month, moderate if they consume 4–14 drinks per week, and heavy if they consume more than 2 standard drinks per day (Dufour 1999).

## GLOSSARY

Antibody: Molecule produced by certain immune cells that recognizes and interacts with foreign substances in the body (i.e., antigens), including viruses and bacteria. Antibodies play an important role in the immune response; each antibody is specific for one antigen. The presence of antibodies to a certain antigen (e.g., hepatitis C virus) indicates that the patient has previously been exposed to this antigen.
Antioxidant: A substance (e.g., vitamin E or glutathione) that can trap or detoxify harmful *free radicals*.
Compensated cirrhosis: Cirrhosis without major life-threatening complications.
Decompensated cirrhosis: Cirrhosis with major life-threatening complications.
Fibrosis: The formation of scar tissue.
Free radical: A highly reactive and often harmful molecule that cannot exist in a free state for a prolonged period; frequently contains oxygen.
Incidence: The number of new cases of a disease that occur within a given time period.
Oxidative stress: An imbalance between oxidants (e.g., *free radicals*) and *antioxidants*; can lead to cell damage.
Prevalence: The number of all cases of a disease existing during a given time period.
Stellate cell: A star-shaped liver cell that serves as the primary storage site for vitamin A compounds and fat molecules; alcohol and factors released from other liver cells can lead to activation of stellate cells, causing them to form scar tissue—a key process in the development of *fibrosis*.
Viral titer: The number of virus particles in a given volume (e.g., one milliliter) of a fluid (e.g., blood); indicates the degree to which the virus can multiply in the organism.
Viremia: The presence of virus particles in the blood, regardless of their number.

## References

[b47-arcr-25-4-245] Colell A (1998). Selective glutathione depletion of mitochondria by ethanol sensitizes hepatocytes to tumor necrosis factor. Gastroenterology.

[b48-arcr-25-4-245] Nanji AA, Hiller-Sturmhöfel S (1997). Apoptosis and necrosis: Two types of cell death in alcoholic liver disease. Alcohol Health & Research World.

